# Improved Safety of New MicroRNA-Regulated Oncolytic Coxsackievirus B3 Observed After Intravenous Administration in Colorectal-Tumor-Bearing Mice

**DOI:** 10.3390/v18010143

**Published:** 2026-01-22

**Authors:** Leslie Elsner, Luisa Hinze, Ahmet Hazini, Lisanne Heimann, Anja Geisler, Babette Dieringer, Karin Klingel, Sophie Van Linthout, Jens Kurreck, Robert Klopfleisch, Henry Fechner

**Affiliations:** 1 Department of Applied Biochemistry, Institute of Biotechnology, Technische Universität Berlin, 13355 Berlin, Germany; l.elsner.1@tu-berlin.de (L.E.); l.heimann.1@tu-berlin.de (L.H.); a.geisler@tu-berlin.de (A.G.); babette.dieringer@tu-berlin.de (B.D.); jens.kurreck@tu-berlin.de (J.K.); 2 Institute of Biochemistry, Charité—Universitätsmedizin Berlin, Corporate Member of Freie Universität Berlin and Humboldt-Universität zu Berlin, 10117 Berlin, Germany; 3 Department of Oncology, University of Oxford, Oxford OX3 7DQ, UK; ahmet.hazini@oncology.ox.ac.uk; 4 Cardiopathology, Institute for Pathology and Neuropathology, University Hospital Tuebingen, 72076 Tuebingen, Germany; karin.klingel@med.uni-tuebingen.de; 5 Berlin Institute of Health Center for Regenerative Therapies (BCRT), Charité–Universitätsmedizin Berlin, Campus Virchow Klinikum (CVK), Föhrer Str. 15, 13353 Berlin, Germany; sophie.van-linthout@charite.de; 6 German Center for Cardiovascular Research (DZHK), Partner Site Berlin–Charité, Oudenarder Straße 16, 13316 Berlin, Germany; 7 Institute of Veterinary Pathology, Freie Universität Berlin, 14163 Berlin, Germany; robert.klopfleisch@fu-berlin.de

**Keywords:** oncolytic virus, coxsackievirus B3, cancer, colorectal carcinoma, microRNAs, PD-H, intravenous application

## Abstract

Oncolytic coxsackievirus B3 (oCVB3) strain PD-H has shown potent oncolytic efficacy and a remarkable safety profile in the treatment of colorectal cancer in vivo after intratumoral (i.t.) injection. In this study, we investigated the safety and efficiency of PD-H following intravenous (i.v.) virus administration. When injected i.v. into Balb/C mice bearing subcutaneous Colon-26 tumors, PD-H led to slightly reduced tumor progression and a significant increase in animal survival, but it also caused multi-organ infection and tissue damage. To improve the safety profile of PD-H, we inserted microRNA target sites (miR-TS) of the heart-specific miR-1, pancreas-specific miR-375, liver-specific miR-122, and brain-specific miR-124 or the tumor-suppressor miR-145 into the genome of PD-H and generated the viruses PD-622TS and PD-145TS. Both viruses replicated similarly and induced cytotoxicity comparable to that of PD-H in the colorectal carcinoma cell lines Colon-26 and CT-26Luc. Their replication was inhibited in HEK293T cells transiently transfected with the cognate microRNAs. In vivo, i.v. administration of PD-145TS and PD-622TS to healthy Balb/C mouse resulted in significantly lower viral titers in the organs of mice and led to significantly less-intense pathological alterations compared to PD-H. PD-622TS injected i.v. into Balb/C mice with CT-26Luc-induced peritoneal carcinomatosis did not induce off-target alterations in normal organs, but it failed to induce a therapeutic effect. These data indicate that PD-H or microRNA-regulated PD derivatives exhibit only limited therapeutic efficacy following i.v. injection in colorectal tumor-bearing mice. However, the newly engineered microRNA-regulated PD-H variants demonstrate improved safety profiles.

## 1. Introduction

Colorectal cancer (CRC) is the third most common cancer in the world, with an incidence of 10.2% and a mortality rate of 9.2% [1]. In Germany, colorectal cancer is the third most common cancer in men and the second most common in women and represents the second leading cause of cancer-related death [2]. In recent years, mortality due to CRC has declined, primarily as a result of the implementation of enhanced screening programs, improved identification of high-risk populations, advancements in surgical techniques, and progress in systemic therapies, including chemotherapy and radiotherapy [3,4,5]. Nonetheless, despite these therapeutic and diagnostic improvements, CRC remains associated with considerable mortality, with five-year survival rates decreasing markedly to approximately 20% among patients with stage III and IV disease [6]. Thus, therapeutic resistance, as well as treatment-related toxicities, continue to pose significant challenges and underscore the urgent need for novel and more effective therapeutic strategies [7]. Oncolytic viruses (OVs) are a novel class of tumor-selective immunotherapeutics that specifically infect and destroy cancer cells while sparing normal tissue. Their antitumor efficacy is mediated by several complementary mechanisms. OVs selectively infect and lyse tumor cells, thereby exerting a direct antitumor effect. Viral infection of tumor cells leads to immunogenic cell death, promoting the release of immunostimulatory signals, including pathogen-associated molecular patterns (PAMPs), damage-associated molecular patterns (DAMPs), tumor-associated antigens (TAAs), and neoantigens. This leads to remodeling of the tumor microenvironment (TME), characterized by fewer immunosuppressive cells, increased pro-inflammatory cytokine production, and a transition from an immunologically “cold” state to a “hot” tumor state, thereby activating innate and adaptive immune responses and ultimately inducing durable and systemic antitumor immunity [8,9].

CVB3 is a single (+)-strand RNA virus from the picornavirus family. Its genome is about 7.5 kb long and contains a single open reading frame, which is flanked at it 5′ end by an internal ribosomal entry site and on its 3′ end by a poly-A-signal [10]. Following the isolation of the virus from a patient approximately 75 years ago, virological studies aimed at elucidating the mechanisms of viral infection, replication, and pathogenesis have led to the discovery and development of a wide range of natural and laboratory-adapted CVB3 strains [11]. Several of these strains have been investigated in recent years for their potential in cancer therapy [12,13,14,15]. These studies demonstrated that the viruses exhibit robust oncolytic activity against breast, pancreatic, lung, endometrial, and colorectal tumors in both xenograft and syngeneic murine models [13,14,15,16,17,18]. However, non-engineered first-generation oCVB3 frequently induced severe and sometimes fatal side effects in vivo. In particular, the viruses infected the heart and the pancreas, representing the most susceptible organs for CVB3 in the murine organism [19], and induced severe organ damage and inflammation [12,16,20,21,22]. To mitigate these off-target effects, microRNA target sites (miR-TS) of heart- and pancreas-enriched microRNAs and/or tumor-suppressor microRNAs were introduced into the genome of oCVB3. This modification reduced—and in some cases completely abolished—viral toxicity, often without compromising the oncolytic activity of the viruses [16,18,20,21,22,23,24].

Our group developed the oncolytic CVB3 strain PD-H [16], a CVB3 variant that possesses a unique receptor tropism for heparan sulfates [12,16], which underlies its broad tumor cell tropism and distinguishes it from other oCVB3 strains that utilize the coxsackievirus and adenovirus receptor (CAR) for cell entry [25,26]. The virus exhibited potent oncolytic activity in murine models of colorectal cancer [12,16] and demonstrated therapeutic efficacy in pancreatic cancer models [17]. Overall, PD-H shows a favorable safety profile. Notably, no off-target infections of the heart or pancreas were observed in immunocompetent mice bearing colorectal or pancreatic carcinomas following intratumoral (i.t.) PD-H administration [16,17].

Previous investigations of oCVB3 have primarily focused on the efficacy and safety of the viruses after i.t. or local administration, and, consequently, studies assessing the therapeutic potential and safety of the viruses following systemic administration are still lacking. In this study, we addressed this gap by evaluating these aspects for the treatment of colorectal carcinomas using the oCVB3 PD-H strain. We demonstrate that i.v. administration of PD-H elicited only a modest therapeutic effect in mice bearing subcutaneous colorectal tumors. Moreover, the i.v. administration of the virus caused tissue damage and inflammation in multiple organs. Using the newly developed, safer, microRNA-regulated PD-H variant PD-622TS, off-target effects were markedly reduced. However, systemic administration of this virus in mice with experimentally induced colorectal peritoneal carcinomatosis failed to result in therapeutic efficacy.

## 2. Materials and Methods

### 2.1. Cell Culture

HEK293T cells were cultured in high-glucose DMEM (Biowest, Darmstadt, Germany) supplemented with 10% fetal calf serum (FCS), 1% penicillin/streptomycin (P/S), 1% L-glutamine, and 1% sodium pyruvate. CaCo-2 cells were grown in high-glucose DMEM (Biowest) supplemented with 10% FCS, 1% P/S, 1% L-glutamine, 1% sodium pyruvate, and 1% non-essential amino acids (NEAA). CHO-K1 cells were cultured in Ham’s F-12 Medium (Thermo Fisher Scientific, Waltham, MA, USA) with 10% FCS and 1% P/S. HeLa cells were maintained in MEM medium (Gibco, Karlsruhe, Germany) containing 5% FCS, 0.02 M HEPES, 1% NEAA, and 1% P/S. The murine colorectal cancer cell line Colon-26 was cultured in RPMI 1640 (c.c.pro, Oberdorla, Germany) supplemented with 10% FCS, 1% P/S, 1% L-glutamine, and 2% sodium pyruvate. The murine colorectal cancer cell line CT-26Luc was maintained in RPMI (c.c.pro) supplemented with 10% FCS, 1% P/S, 1% L-glutamine, 2% sodium pyruvate, and 1% NEAA.

### 2.2. MicroRNA Expression Analysis

Total RNA from colorectal tumor cells or mouse tissues was isolated by using TRizol reagent (Thermo Fisher Scientific, Waltham, MA, USA) according to the manufacturer’s instructions and reverse-transcribed by using the High-Capacity cDNA Reverse Transcription Kit (Applied Biosystems, Foster City, CA, USA). Expression levels of miR-375 (assay ID: 000564), miR-1 (assay ID: 002222), miR-124 (assay ID: 003188_mat), miR-122 (assay ID: 002245), miR-143 (assay ID: 002249), miR-145 (assay ID: 002278), miR-195 (assay ID: 000494), and miR-339 (assay ID: 002257) were determined by utilizing the TaqMan gene expression master mix and specific TaqMan gene expression assays from Life Technologies according to the manufacturer’s instructions. The applied TaqMan assays detect the respective mature microRNAs of both mice and humans. Real-time PCR was performed by using a CFX96 Real-Time System combined with a C1000Thermal Cycler (Bio-Rad Laboratories, Hercules, CA, USA). The data were analyzed using the ΔΔCT method. The results were normalized against U6 snRNA (assay ID: 001973).

### 2.3. Cloning of miR-TS into PD-H cDNA

pJet-CVB3-PD-H [16] containing the full-length cDNA of PD-H served as a plasmid for the cloning of miR-TS. The fragment that was inserted into pJet-CVB3-PD-H to generate pJet-CVB3-PD-145TS consists of three tandem repeats of miR-145TS. The fragment that was inserted into pJet-CVB3-PD-H to generate pJet-CVB3-PD-622TS consists of two tandem repeats of miR-375TS, one miR-122TS, one miR-124TS, and two tandem repeats of miR-1TS. The miR-TS fragments were obtained via gene synthesis (Thermo Fisher Scientific). Specific primers for amplifying the inserts were designed using the In-Fusion Primer Design Tool (Takara Bio USA, Mountain View, CA, USA). The amplified PCR fragments were inserted into the plasmid pJet-CVB3-PD-H, and the final constructs were assembled using the In-Fusion HD Cloning Kit (Takara Bio), according to the manufacturer’s instructions.

### 2.4. Dual-Luciferase Reporter Assay

A total of *2*.5 × 10^5^ Colon-26 cells and 1.1 × 10^5^ HeLa cells were seeded into 24-well plates. After 24 h, cells were co-transfected with 100 ng of the dual-luciferase reporter plasmid psiCHECK2-miR-375TS [19] containing miR-375TS within the 3’UTR of Renilla luciferase gene and 700 ng of either the miR-375-expressing plasmid pCMV-MIR-375-GFP or the miR-216a-expressing plasmid pCMV-MIR-216a-GFP using Lipofectamine™ 3000 (Thermo Fisher Scientific). Both plasmids were purchased from OriGene (OriGene Technologies Inc., Rockville, MD, USA). The following day, Firefly and Renilla luciferase activity were measured on a Tristar5 plate reader (Berthold Technologies, Bad Wildbad, Germany) via the dual-luciferase reporter assay (Promega GmbH, Walldorf, Germany) according to the manufacturer’s instructions.

### 2.5. Generation of miR-TS Containing PD-H

For the generation of the recombinant virus PD-H, CHO-K1 cells were transfected with pJet-CVB3-PD-H; for the generation of PD-145TS, CaCo-2 cells were transfected with pJet-CVB3-PD-145TS; and for the generation of PD-622TS, HEK-293T cells were transfected with pJet-CVB3-PD-622TS. Cells were seeded into 6-well plates and transfected at 70–80% confluence with 2.5 µg of the respective plasmids. Transfections were performed using PEImax (Polysciences Europe GmbH, Hirschberg an der Bergstraße, Germany) for HEK-293T and CHO-K1 cells and Lipofectamine™ 3000 (Thermo Fisher Scientific) for CaCo-2 cells. Then, 72 h post-transfection, cells were lysed via three freeze–thaw cycles. Cell debris was removed via centrifugation, and the resulting supernatant was used to determine viral titers via plaque assay to HeLa cells. To obtain higher titers, the virus PD-622TS was amplified in HEK293T cells, and PD-H and PD-145TS were amplified in CHO-K1 cells. Purification and concentration for in vivo experiments were carried out with sucrose gradient centrifugation, as described earlier [19].

### 2.6. Determination of Virus Titers Using the Plaque Assay

Virus plaque assay was carried out as described previously [20]. Briefly, HeLa cells were seeded as a confluent monolayer in 24-well plates and cultured for 24 h. Subsequently, a tenfold serial dilution of the virus samples was prepared in PBS. The culture medium of the HeLa cells was carefully removed, and the cells were overlaid with 300 µL of the respective virus dilution. After incubation for 30 min, the viral suspension was aspirated, and the cells were covered with 500 µL of 3.2% BD-Difco NobleAgar (Thermo Fisher Scientific, Waltham, MA, USA) containing MEM (Gibco, Karlsruhe, Germany). Then, 72 h after viral infection, staining was carried out with Tetrazoliumbromid-Iodnitrotetrazoliumchlorid solution (both VWR International GmbH, Darmstadt, Germany).

### 2.7. Viral Silencing in MicroRNA-Transfected HEK293T Cells

A total of 1.8 × 10^5^ HEK293T cells were seeded into 24-well plates. Twenty-four hours after seeding, cells were transfected using PEImax (Polysciences Inc., Warrington, PA, USA) with 1 µg of the plasmids pCMV-mir375-GFP, pCMV-mir216a-GFP, pCMV-mir124-GFP, pCMV-mir145-GFP (all Origene Technologies, Rockville, MD, USA), Vec-mir1-2, and Vec-mir122 [27], which express miR-375, miR-216a, miR-124, miR-145, miR-1, and miR-122, respectively. Then, 48 h after transfection, cells transfected with miR-145 were infected at a multiplicity of infection (MOI) of 0.01 with PD-145TS, and cells transfected with miR-375, miR-1, miR-122, or miR-124 were infected with PD-622TS. Cells transfected with miR-216a were infected with both viruses and served as a control. Then, 24 h post-infection, cells were disrupted by three freeze–thaw cycles, cell debris was removed via centrifugation, and the virus-containing supernatant was used for determination of virus titers using plaque assay in HeLa cells.

### 2.8. Virus Growth Curves

Viral growth curves were generated to analyze virus replication in Colon-26 and CT-26Luc cells. Cells were seeded into 96-well plates to reach approximately 80% confluence after 24 h. On the following day, cells were infected with 100 µL of virus suspension at MOI of 0.1. After 1 h of incubation, the virus solution was removed, and cells were overlaid with 200 µL of fresh medium. At 0, 8, 24, and 48 h post-infection, cells were subjected to three freeze–thaw cycles. Cell debris was removed via centrifugation, and the supernatant was collected for determination of viral titers.

### 2.9. Cell Viability Assay

Cell viability was assessed using the Cell Proliferation Kit (XTT) (Promega GmbH, Walldorf, Germany) according to the manufacturer’s instructions. Colon-26 and CT-26Luc cells were seeded into 96-well plates to reach approximately 80% confluence after 24 h. The following day, cells were infected using different MOIs. Absorbance was measured 24 and 48 h post-infection using a TriStar^2^ LB 942 Multimode Microplate Reader (Berthold Technologies). Cells treated with 50 µL of 5% Triton X-100 served as a control for complete cell lysis.

### 2.10. Quantitative Analysis of CVB3 RNA Levels in Pancreas Tissue via qRT-PCR

Due to tissue-specific methodological limitations that precluded reliable plaque assay readouts, viral load in pancreas samples was determined using RT-qPCR. Quantification of viral RNA was carried out as described previously [12]. Briefly, total RNA was isolated from pancreas tissue using TRIzol™ Reagent (Thermo Fisher Scientific) according to the manufacturer’s instructions. Tissue homogenization was performed using Pellet Pestles. RNA samples were stored at −80 °C until further use. For cDNA synthesis, viral RNA was reverse-transcribed using Multiscribe™ Reverse Transcriptase (Applied Biosystems, Foster City, CA, USA). Quantification of CVB3 RNA was performed via qPCR using the SsoFast™ EvaGreen^®^ Supermix (Bio-Rad Laboratories, Hercules, CA, USA) and CVB3-specific primers. Based on the Ct values of the CVB3 standards, CVB3 RNA copy numbers were calculated for each sample.

### 2.11. Syngeneic Subcutaneous Colon-26 Cancer Mouse Model

All animal procedures were approved by the State Office of Health and Social Affairs, Berlin, Germany (reference number G0048/18 for 2.11 and 2.12, reference number E0023/23 for 2.13), and conducted in strict accordance with the Animal Research: Reporting of In Vivo Experiments (ARRIVE) guidelines to ensure scientific rigor and reproducibility.

A syngeneic subcutaneous Colon-26 cancer mouse model was established using 6-week-old female BALB/c mice (Charles River Laboratories, Sulzfeld, Germany) in accordance with institutional guidelines for animal care. A total of 5 × 10^5^ Colon-26 cells in 100 µL total volume were injected subcutaneously into the right flank of each mouse. When tumors reached an approximate diameter of 0.5 cm, tumor-bearing mice were randomized into experimental groups and i.v. injected via the jugular vein with either 1 × 10^7^ PFU of PD-H (*n* = 11) or PBS (*n* = 8) in a total volume of 70 µL. For biodistribution studies, 6 PD-H-infected mice and 4 PBS-treated control mice were sacrificed 3 days after virus injection. Tumors and organs were harvested. Each tumor was divided into two parts, and two pieces were taken from each organ: one part was snap-frozen in liquid nitrogen and stored at −80 °C for subsequent analysis of viral content, while the other part was fixed in 4% paraformaldehyde for histological examination.

The remaining animals (5 PD-H–infected and 6 PBS-treated mice) were monitored for survival until day 32 after tumor cell injection, which represented the end of the observation period for Kaplan–Meier survival analysis. Tumor size was measured, and tumor volume was calculated using the ellipsoid volume formula: 0.5 × L × W^2^.

### 2.12. Balb/C Model for Virus Biodistribution Studies

For the establishment of a safety profile and to analyze the viral biodistribution, 6-week-old female Balb/C mice (Charles River Laboratories) were randomly divided into three groups and i.v. injected via the jugular vein with 1 × 10^7^ PFU of PD-H (*n* = 8), PD-145TS (*n* = 7), or PD-622TS (*n* = 8) in a total volume of 70 µL. Mice were sacrificed 3 days post-injection, and organs were harvested. Each organ was divided into two parts, and two pieces were taken from each organ: one part was snap-frozen in liquid nitrogen and stored at −80 °C for subsequent analysis of viral titers, while the other part was fixed in 4% paraformaldehyde for histological examination. Blood was collected at the time of sacrifice via cardiac puncture, allowed to clot at room temperature, and centrifuged at 2000× *g* for 5 min to obtain serum, which was stored at −80 °C until further analysis.

### 2.13. Syngeneic CT-26Luc Peritoneal Carcinomatosis Cancer Mouse Model

A syngeneic CT-26Luc peritoneal carcinomatosis cancer mouse model was established using 6-week-old female BALB/c mice (Janvier Labs, Le Genest-Saint-Isle, France) in accordance with institutional guidelines for animal care. A total of 1 × 10^5^ CT-26Luc cells were injected intraperitoneally (i.p.) into Balb/c mice in 100 µL total volume. Two days after cell injection, mice were randomized based on baseline bioluminescence signal intensity into experimental groups and injected i.v. via the tail vein with either 2 × 10^7^ PFU PD-622TS (*n* = 6) or PBS (*n* = 5) in 70 µL total volume. Bioluminescence imaging was carried out on days 2, 6, 10, and 13 after tumor cell injections, as described in 2.14. Mice were sacrificed 13 days after tumor cell injection, and tumors, organs, and blood were collected for determination of viral titers.

### 2.14. In Vivo Bioluminescence Imaging

Bioluminescence imaging (BLI) was conducted using the NightOwl LB 981 system (Berthold Technologies). Mice were anesthetized with isoflurane (Abbott GmbH, Wiesbaden, Germany) and administered 150 mg/kg of D-luciferin (Biosynth, Staad, Switzerland) dissolved in sterile PBS via i.p. injection. Tumor growth was monitored and semi-quantified using IndiGoVersion 2.0.5 (Berthold Technologies).

### 2.15. Histopathological Analysis

Formalin-fixed, paraffin-embedded tissue samples were sectioned at 5 µm and stained with hematoxylin and eosin (H&E) for histopathological evaluation of tissue integrity, cell destruction, and inflammation. To quantify organ damage, comprising cell necrosis, inflammation, and scarring, we applied a score of 0 to 4 (score: 0, no lesion; 1, minimal lesion; 2, mild lesion; 3, moderate lesion; and 4, severe lesion).

### 2.16. Immunohistochemical Analysis

Expression CVB3 VP1 was detected via immunohistochemistry using Cox mAB 31A2 antibody (Mediagnost, Reutlingen, Germany). Briefly, paraffin sections were pretreated with heat antigen retrieval (citrate buffer/microwave), a Mouse-on-mouse KIT from DAKO, and a goat anti-mouse secondary antibody. The signal was visualized using DAB. An evaluation was performed to quantify the strength of VP1 immunoreactivity. The evaluation was based on the assessment of signal intensity observed in organ slides. The following scoring system was used: 0 (no immunoreactivity), 1 (minimal, with a few single cells with immunoreactivity), 2 (mild, with several single cells with immunoreactivity), 3 (moderate, with a few groups of cells with immunoreactivity), and 4 (severe, with numerous cells and groups of cells exhibiting immunoreactivity).

### 2.17. Statistical Analysis

Statistical analyses were performed using GraphPad Prism version 8.2 (GraphPad Software, Boston, MA, USA). For the in vitro experiments, a two-tailed unpaired Student’s t-test was applied to evaluate statistical significance. For the analysis of animal experiments, the Mann–Whitney U test was used. For the statistical evaluation of virus biodistribution (virus-positive vs. virus-negative animals), Fisher’s exact test was applied. Survival was analyzed using Kaplan–Meier survival curves, and differences between groups were assessed using the log-rank (Mantel–Cox) test. Data are presented as mean ± SEM. Differences were considered statistically significant at *p* < 0.05.

## 3. Results

### 3.1. Intravenous Application of PD-H Has Limited Therapeutic Efficiency in BalB/C Mice Bearing Subcutaneous Colorectal Tumors and Results in Viral Infection of Normal Organs

**Figure 1:**
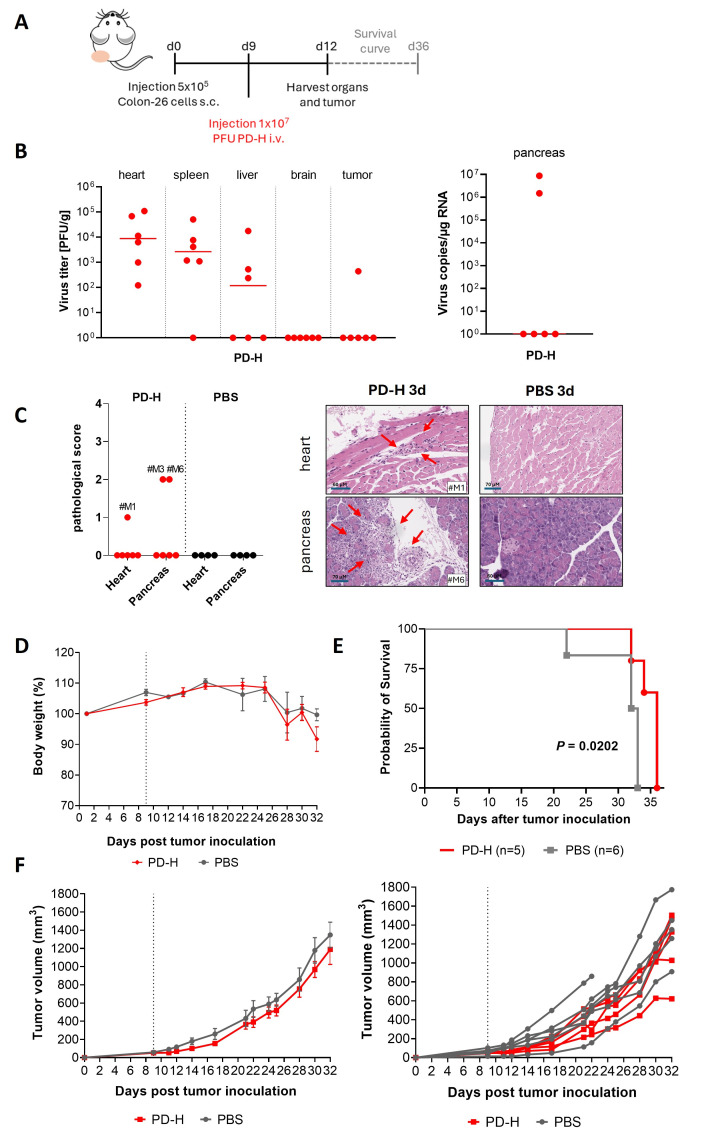
Effectiveness and safety of PD-H in a syngeneic subcutaneous colorectal cancer model after i.v. virus administration. (**A**) Experimental timeline. Colon-26 cells were subcutaneously inoculated into the right flank of Balb/C mice. When the tumor reached a diameter of ~0.5 cm, 1 × 10^7^ PFU of PD-H (*n* = 11) or PBS (*n* = 8) was i.v. injected into the vena jugularis. (**B**) Analysis of virus titers in organs and tumors 3 days after PD-H injection. (**C**) Histological examination of mouse organs on day 3 after PD-H injection. Left, score of pathological alteration in the pancreas and the heart; Right, H&E-stained tissue sections from PD-H–treated animals, showing the most pronounced organ damage observed in this experiment (heart score, 1; pancreas score, 2), and from PBS-injected control animals. #M1 = mouse 1, #M3 = mouse 3, and #M6 = mouse 6. Arrows indicate pathological alterations. Note: In panels B and C, organs from all PD-H–treated mice (*n* = 6) and control mice (*n* = 4) are analyzed. (**D**) Bodyweight development. (**E**) Kaplan–Meier survival curve, indicating significance for PD-H vs. PBS-group *p* = 0.0202. (**F**) Tumor volumes. Left, tumor volumes are shown as means ± SEM. Right, Tumor volumes are shown for each animal. Note: in D-F, *n* = 5 (PD-H) and *n* = 6 (PBS) animals were investigated.

Recently, we demonstrated the potent oncolytic activity of the CVB3 variant PD-H in mice following i.t. injection in both subcutaneous xenograft and syngeneic colorectal tumor models [12,16]. To explore whether colorectal carcinomas can also be treated using i.v. administrated PD-H, we injected PD-H-sensitive Colon-26 tumor cells into the flanks of Balb/C mice. Once the tumors reached a volume of approximately 50 mm^3^, the mice received an i.v. injection of either 1 × 10^7^ PFU PD-H (*n* = 11) or PBS (*n* = 12) via the jugular vein (Figure 1A). Six animals each from the virus-infected and control groups were investigated 3 days later for virus infection and pathological alterations using plaque assays and histopathological investigation, respectively. Among the virus-infected animals, PD-H was detected in the tumor of one animal, whereas it was found in the hearts of all the animals, in the spleens of five, in the livers of three, and in the pancreas in two animals. The brain was virus-free. The PD-H titers were generally low to moderate (Figure 1B). Inflammation and tissue damage were detected in the pancreas in two of the PD-H-infected animals and in the heart in one, but it was not found in other organs (spleen, liver, and brain) in the animals in this group nor in the animals in the control group. The severity of pathological changes ranged from mild to moderate (Figure 1C). Five animals infected with PD-H and six control animals were further investigated until day 36 after tumor cell injection. From day 24 onwards, tumor-related disease progression led to a continuous decline in body weight, which was comparable between both treatment groups (Figure 1D). Animal survival was significantly improved in the group treated with PD-H compared to the control group (Figure 1E). Compared to the control animals, we also observed slightly reduced tumor progression in the PD-H-treated animals starting 3 days after virus injection and continuing until the end of the study period. However, differences between the PD-H-injected and control animals did not reach statistical significance (Figure 1F). We also analyzed the organs of mice investigated over a long period for pathological alterations. No pathological changes were detected in the heart, pancreas, spleen, liver, or brain in either the PD-H-infected group or the control group. 

Collectively, these data demonstrate that i.v. delivery of PD-H confers limited oncolytic activity against colorectal carcinoma while promoting early productive infection across multiple murine organs.

### 3.2. Evaluation of Tissue-Specific and Tumor Suppressor MicroRNAs in Normal Tissues and Colorectal Carcinoma Cell Lines

**Figure 2:**
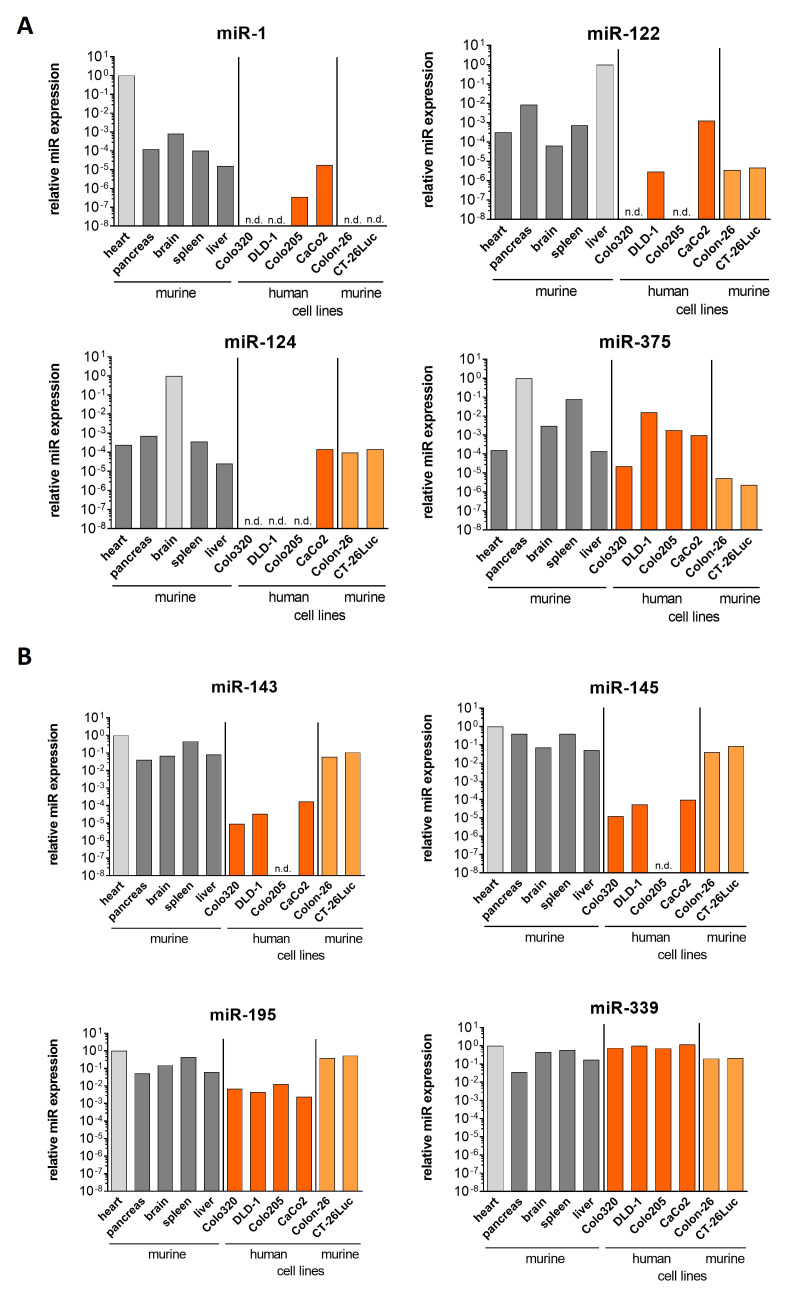
Expression of microRNAs in murine organs and human and murine colorectal cancer cell lines. (**A**) Expression of tissue-specific microRNAs. (**B**) Expression of tumor-suppressor microRNAs. Expression levels were determined via qRT-PCR. Each microRNA expression level was normalized against the level of endogenous U6 snRNA expression. The expression levels are shown relative to the highest microRNA expression level found in a murine organ, which was set to 10^0^. n.d. = not detected. Note: The applied TaqMan assays detect the respective mature microRNAs of both mice and humans. For analysis, the same assay chemistry was used to enable comparison of microRNA expression levels in murine and human samples.

Based on the data obtained from the experiments shown in Figure 1, which demonstrate that both the safety of PD-H and its tumor-targeting capability are limited, we prioritized the improvement of the safety profile of PD-H over the enhancement of its tumor specificity in the subsequent investigations. This decision was made following a “safety-first” strategy, based on the principle that viral safety should be firmly established before attempts are made to increase therapeutic efficacy. Thus, next, we developed a microRNA-regulated PD-H designed to de-target virus infection from the heart, pancreas, brain, and liver, as these organs represent major target tissues for CVB3 in humans [28,29,30,31]. First, we identified microRNAs that are highly expressed in all four organs but expressed at low levels in colorectal tumor cells. To this end, we analyzed four tumor-suppressive microRNAs (miR-143, miR-145, miR-195, and miR-339) [32,33,34,35] and four microRNAs reported to be highly expressed in the heart (miR-1), pancreas (miR-375), liver (miR-122), and brain (miR-124) [36]. All four tissue-specific microRNAs showed very high expression levels in their respective target organs. In contrast, their expression in other murine organs was markedly lower—by factors ranging from 10- to 10^5^-fold. Similarly, in both murine and human colorectal tumor cells, the four microRNAs were expressed at very low levels or were, in some cases, undetectable (Figure 2A). The four tumor-suppressor miRNAs were highly expressed across all analyzed organs. miR-143 and miR-145 exhibited similar expression profiles. In human colorectal cancer cell lines, their expression levels were generally at least 10^3^-fold lower than in murine organs. In contrast, in the two murine colorectal tumor cell lines CT-26Luc and Colon-26, the expression level of miR-143 was comparable to that in the murine pancreas, brain, and liver but approximately 5- to 10-fold lower than in the heart and spleen. The miR-145 showed a similar expression pattern to miR-143, except that its expression in the pancreas was also 5- to 10-fold lower than in CT-26Luc and Colon-26 cells. A moderately lower expression (10- to 100-fold) in human colorectal tumor cell lines compared to murine organs was also observed for miR-195. However, the expression of this microRNA in CT-26Luc and Colon-26 cells was similar to, or even slightly higher than, that in murine organs. For miR-339, no marked differences were detected between its expression in murine organs and in human or murine colorectal cancer cell lines (Figure 2B).

Taken together, these results show that all four selected tissue-specific microRNAs appear to be suitable regulators for PD-H. Among the tumor-suppressor microRNAs tested, miR-145 proved to be the most effective candidate.

### 3.3. Inhibition of PD-145TS and PD-622TS Replication by Corresponding miRs

**Figure 3:**
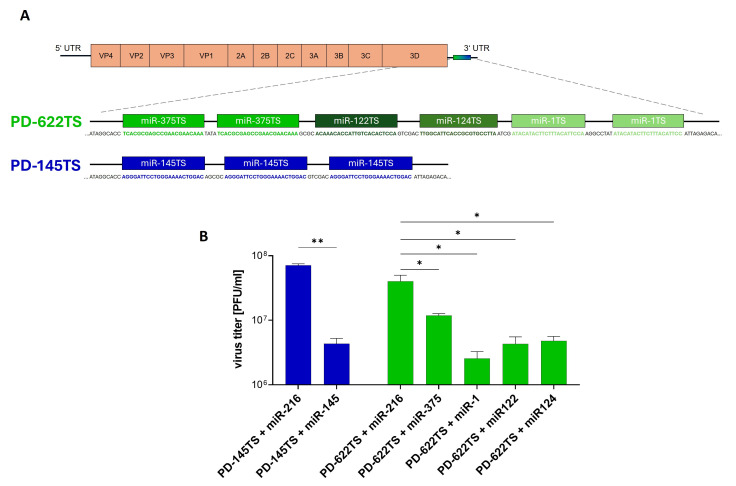
Inhibition of PD-622TS and PD-145TS by microRNAs. (**A**) Scheme of PD-622TS and PD-145TS genomes. Note that the number 622 in the name PD-622TS indicates the sum of the microRNAs whose target sites were inserted into the virus. This was performed to make the name easier to read. The miR-TS were inserted into the 3′ UTR of the viral genome immediately downstream of the 3D polymerase encoding sequence. (**B**) Virus inhibition assay. HEK293T cells were seeded into 24-well plates. Then, 24 h later, the cells were transfected with plasmids expressing the miR-216, miR-145, miR-375, miR-1, miR-122, or miR-124 and incubated for a further 48 h. Subsequently, cells were infected with PD-145TS or PD-622TS at an MOI of 0.01 and incubated for further 24 h. Virus titers were determined using the plaque assay in HeLa cells. Data are presented as mean ± SEM (*n* = 3). Significance levels: * *p* < 0.05; ** *p* < 0.01.

After selection of the miR, the PD-H derivatives PD-145TS and PD-622TS were generated. PD-145TS was constructed by inserting three copies of the miR-145 target sequence (miR-145TS) into the 3′ UTR of the PD-H genome, whereas PD-622TS was generated by inserting one copy each of miR-122TS and miR-124TS, and two copies each of miR-1TS and miR-375TS, into the 3′ UTR of PD-H (Figure 3A). To determine the functionality of miR-TS, a virus inhibition assay was performed. HEK-293T cells were transfected with plasmids expressing miR-1, miR-122, miR-124, miR-145, miR-375, or the control, miR-216, and, 48 h later, infected with either PD-145TS or PD-622TS at an MOI of 0.01. Virus titers were determined 24 h post-infection. Compared to the miR-216-transfected control cells, the titers of PD-145TS and PD-622TS were markedly reduced in cells expressing the corresponding miRs (Figure 3B).

These findings indicate that the miR-TSs within the viral genomes were effectively recognized by their cognate microRNAs, resulting in inhibition of viral replication.

### 3.4. PD-145TS and PD-622TS Replicate and Induce Cell Lysis in Colon-26 and CT-26Luc Cells in a Manner Similar to PD-H

**Figure 4:**
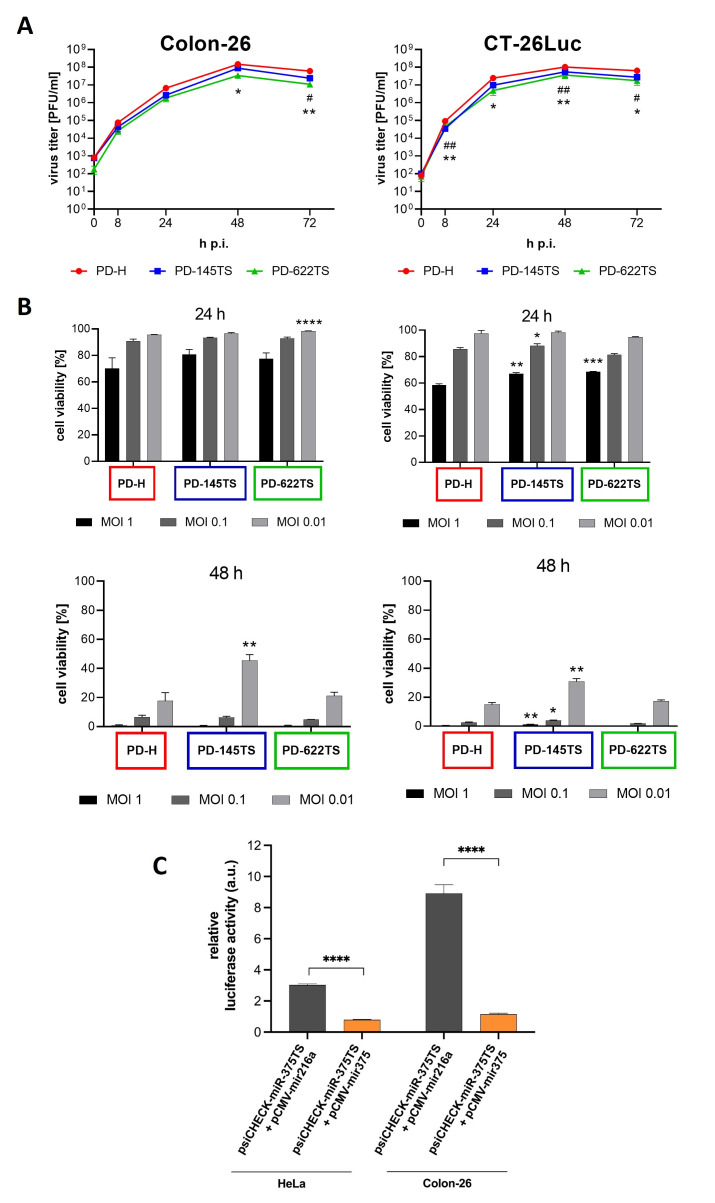
Virus growth kinetics and cytotoxicity of PD-145TS and PD-622TS in murine colorectal carcinoma cell lines. (**A**) Virus growth kinetics. Cells were infected with PD-H, PD-145TS, and PD-622TS at MOI 0.1. Virus titers were determined 0, 8, 24, 48, and 72 h after infection via plaque assay in HeLa cells. Mean values ± SEM (*n* = 3) are shown. Significance—PD-H vs. PD-622TS: * *p* < 0.05 and ** *p* < 0.01. Significance—PD-H vs. PD-145TS: ^#^ *p* < 0.05 and ^##^ *p* < 0.01. (**B**) Cell viability. Cells were infected with indicated viruses at the indicated MOIs and cell viability was determined. Cell viability was measured using the XTT assay 24 h and 48 h after infection. Cell viability was normalized to untreated cells (=100%). Mean values ± SEM (*n* = 3) are shown. Significance compared to PD-H: * *p* < 0.05, ** *p* < 0.01., *** *p* < 0.001, and **** *p* < 0.0001. (**C**) Inhibition of miR-375TS containing luciferase reporter by miR-375 in Colon-26 cells. Colon-26 cells were co-transfected with either miR-375 or the control miR-216, together with a luciferase reporter plasmid containing miR-375TS in its 3′ UTR, and luciferase activity was measured 24 h later. Mean values ± SEM (*n* = 3) are shown. Significance: **** *p* < 0.0001.

As an initial step toward evaluating PD-145TS and PD-622TS in a syngeneic colorectal cancer mouse model, we initially characterized their replication kinetics and cytotoxic potential in vitro using the murine colorectal cancer cell lines Colon-26 and CT-26Luc. Virus growth was assessed over a 72 h period following infection with PD-145TS, PD-622TS, or PD-H (control) at an MOI of 0.1. The viral titers were determined via the plaque assay using HeLa cells. Both PD-145TS and PD-622TS replicated to an extent similar or only slightly inferior to PD-H in Colon-26 and CT-26Luc cells (Figure 4A). To assess viral cytotoxicity, both cell lines were infected with PD-145TS, PD-622TS, or PD-H at MOIs of 0.01, 0.1, and 1, and cell viability was measured using the XTT assay after 24 and 48 h. Also, compared to PD-H, both PD-145TS and PD-622TS exhibited similar or only slightly reduced cytotoxicity in the two colorectal cancer cell lines (Figure 4B). The almost-unaffected replication and cytotoxicity of PD-145TS in both colorectal cancer cell lines constitute a rather surprising finding given the relatively high expression of miR-145 in both cell lines (Figure 2B). This raised the question of whether the miR signaling pathway might be disrupted in these cells, thereby preventing the inhibition of PD-145TS by the miR-145. (Figure 2B). To investigate this possibility, an miR-mediated indicator gene-silencing assay was performed. Colon-26 cells were co-transfected with either miR-375 or the control miR-216, together with a luciferase reporter plasmid containing the miR-375TS within the 3′ UTR of the luciferase cDNA. Measurement of luciferase activity 24 h after indicator gene transfection revealed a strong suppression of luciferase expression in cells transfected with the miR-375 (Figure 4C), indicating that the microRNA pathway was functionally active in Colon-26 cells.

In summary, these results show that both Colon-26 and CT-26Luc cells are highly susceptible to PD-145TS and PD-622TS.

### 3.5. PD-145TS and PD-622TS Cause Fewer Organ Infections and Pathological Alterations than PD-H After i.v. Virus Administration In Vivo

**Figure 5:**
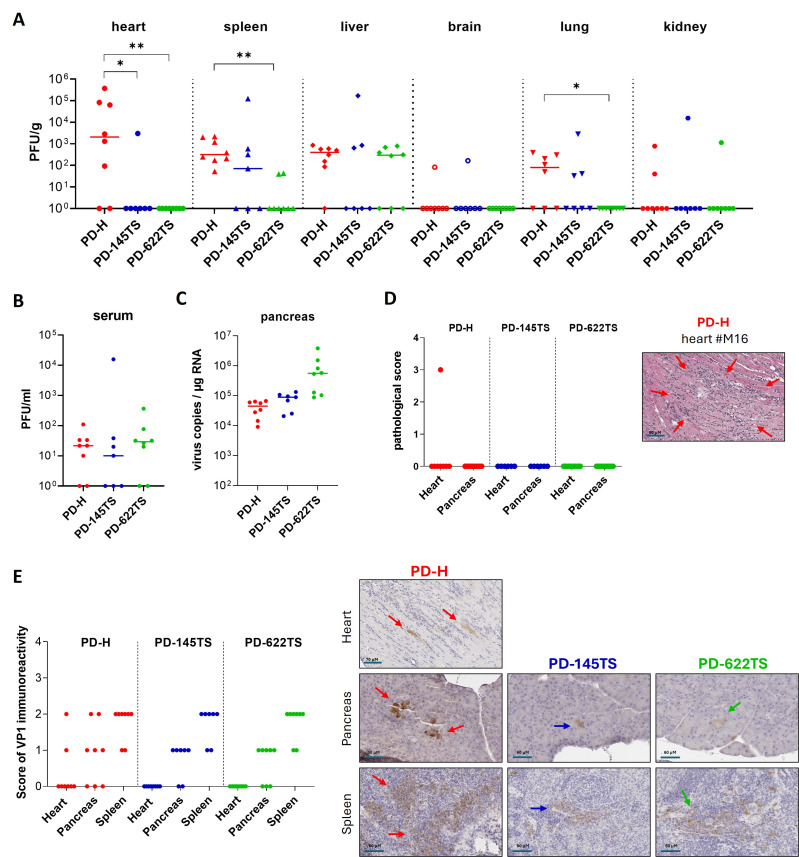
Biodistribution of CVB3 in organs after i.v. injection. A total of 1 × 10^7^ PFU of PD-H, PD-145TS, or PD-622TS was injected i.v. into the jugular vein in Balb/C mice, and investigations were conducted 3 days later (PD-H and PD-622TS, each *n* = 8; PD-145TS, *n* = 7). (**A**) The viral titers were determined using the plaque assay in HeLa cells. For the statistical evaluation of virus biodistribution (PD-H vs. PD-145TS, PD-H vs. PD-622TS, and PD-145TS vs. PD-622TS), Fisher’s exact test was applied. Significance: * *p* < 0.05; ** *p* < 0.01. (**B**) Biodistribution of the viruses in serum. There were no statistically significant differences between groups according to Fisher’s exact test. (**C**) Detection of genomic RNA of the three viruses in the pancreas via RT-PCR. (**D**) Histological examination of mouse organs. Left diagram: Tissue slides of heart and pancreas were stained with H&E, and the degree of pathological alteration was scored (range 0–4); right image: Slide of H&E-stained tissues of the heart of animal #M16 from the group treated with PD-H. Arrows show area with cell damage and inflammation. (**E**) Immunohistochemical examination of mouse organs. Left diagram: CVB3 VP1 immunoreactivity was determined and scored in tissue slides of heart, pancreas, and spleen; right images: representative slides with CVB3 VP1 immunoreactivity. Arrows show cells with immunoreactivity.

To verify the safety of PD-145TS and PD-622TS in vivo after i.v. injection, 1 × 10^7^ PFU of the viruses or PD-H was administered to Balb/C mice via jugularis vein injection. The animals (*n* = 7 to 8 per group) were sacrificed 3 days later. Virus titers in the organs were measured using the plaque assay in HeLa cells or via real-time RT-PCR (only for the pancreas), the expression of viral VP1 was detected via immunohistochemistry (IHC) using a VP1-specific antibody, and organ samples were screened for pathological alteration after being stained with H&E. On day 3 post-infection, PD-H was detected in all organs (heart, spleen, liver, brain, lungs, kidneys, and pancreas) and in the serum. In the heart, spleen, liver, lungs, pancreas, and serum, most animals showed evidence of PD-H infection. In contrast, viral presence was detected in the brain in only one animal and in the kidneys in two animals. The PD-H titers were generally low; moderate virus loads were detected exclusively in the heart in three animals. In comparison to PD-H, i.v. PD-622TS administration significantly reduced the number of animals with infections in the heart, spleen, and lungs. In contrast, PD-145TS only exhibited this effect in the heart (Figure 5A–C). Histopathological examination revealed distinct signs of myocarditis in one PD-H-infected animal. All other organs of this animal, as well as all the organs examined in the remaining animals, were free from pathological alterations (Figure 5D). IHC of the heart revealed CVB3 VP1 positivity in two animals infected with PD-H, whereas VP1 could not be detected in the PD-145TS- and PD-622TS-infected animals. In the pancreas, most of the animals infected with the three viruses were VP1-positive, but compared to PD-145TS and PD-622TS, the signal intensity tended to be stronger in the PD-H group. The spleens of all the mice infected with all three viruses showed VP1 immunoreactivity, and the strength of the signal was similar between the groups (Figure 5E).

These results show that both PD-145TS and PD-622TS possess enhanced safety relative to the parental virus PD-H after i.v. administration. Among the two miR-TS variants, PD-622TS displayed slightly enhanced safety profiles with fewer organ infections. Therefore, this virus was selected for further in vivo studies.

Intravenously injected PD-622TS showed a good safety profile but lacked therapeutic efficiency in a model of CT-26Luc-induced peritoneal carcinomatosis

**Figure 6:**
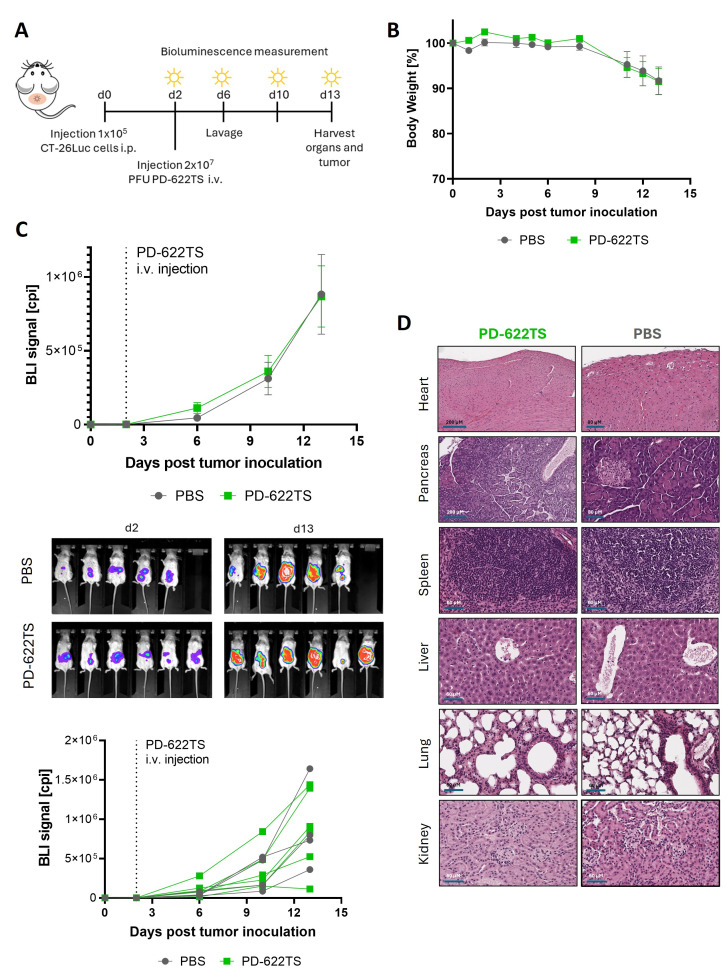
Effectiveness and safety of PD-622TS in a syngeneic colorectal cancer model of peritoneal carcinomatosis following i.v. virus administration. (**A**) Experimental timeline: CT-26Luc cells were injected i.p. into Balb/C mice. Two days later, 2 × 10^7^ PFU of PD-622TS (*n* = 6) or PBS (*n* = 5) was i.v. injected via the tail vein. (**B**) Bodyweight development. (**C**) Tumor development. Development of tumor size was evaluated using BLI. Top diagram*:* BLI on days 6, 10, and 13 after CT-26Luc inoculation into mice. Middle: Representative BLI images of the animals on days 2 and 13 after CT-26Luc administration. Bottom: Tumor growth curves for each animal. (**D**) Histological examination of murine tissues: Representative H&E-stained sections of various organs collected on day 13.

To investigate whether i.v.-injected PD-622TS inhibits colorectal tumor growth in vivo, we employed a model of peritoneal carcinomatosis. This model more accurately reflects an advanced clinical stage of human colorectal cancer compared to the subcutaneous tumor murine model used in our initial investigations. Furthermore, we recently demonstrated that PD-H equipped with miR-375TS showed therapeutic efficacy after i.p. administration in this model [16], enabling us to compare the efficacy of i.p.- and i.v.-administered PD-H variants. For this purpose, 1 × 10^5^ CT-26Luc cells were i.p. injected into mice. Two days later, the mice were injected i.v. via the tail vein with 2 × 10^7^ PFU of PD-622TS (*n* = 6) or with PBS (*n* = 5). Animals were monitored for 13 days after tumor cell injection (Figure 6A); at this point, all animals had to be sacrificed for animal welfare reasons. Animal bodyweight remained stable up to day 8 after tumor cell application and then dropped down continuously up to the end of the investigation (Figure 6B). Bioluminescence measurement revealed that treatment with PD-622TS did not result in a reduction in tumor growth relative to the control mice (Figure 6C). Histological investigations of the heart, the pancreas, the brain, the liver, and the lungs on day 13 did not reveal organ damage or inflammation in any of the six PD-622TS-treated mice or in any of the five control mice (Figure 6D).

Collectively, these findings demonstrate that PD-622TS exhibits a favorable safety profile but does not confer a therapeutic benefit in the treatment of colorectal peritoneal carcinomatosis after i.v. delivery.

## 4. Discussion

The i.v. administration of OVs represents an attractive strategy in cancer virotherapy, as it offers several advantages over local delivery. Systemic application enables broad viral dissemination and access to multiple or metastatic tumor sites that would otherwise be inaccessible through intratumoral (i.t.) injection [37]. This is particularly relevant for tumors located in internal organs as well as metastatic or recurrent lesions, since systemic delivery can reach all tumor manifestations [38]. In addition, i.v. delivery constitutes a non-invasive and clinically practical approach that can be repeatedly applied and readily combined with other therapeutic modalities such as chemotherapy or immune checkpoint inhibition.

In this study, we investigated the therapeutic potential and biosafety of the oCVB3 variant PD-H and its newly engineered microRNA-regulated derivatives PD-622TS and PD-145TS after systemic administration in mice. While PD-H has previously demonstrated potent and selective oncolytic activity in syngeneic Colon-26 cancer models after i.t. delivery [16], here, we found that its efficacy was markedly lower upon i.v. application. Something similar was observed for the microRNA-regulated PD-622TS, which showed no therapeutic effect after i.v. application in a model of CT-26Luc-induced peritoneal carcinomatosis, in which we had previously observed oncolytic activity after i.p. administration of the microRNA-regulated PD-H variant PD-375TS [16]. Little or no therapeutic effect in vivo after i.v. application of OV has already been observed in other studies, including in a study involving a recombinant vesicular stomatitis virus or in investigations involving oncolytic reovirus [39,40]. Although other in vivo [41,42,43] and patient studies [44] have also reported therapeutic success following i.v. administration of OV, these data show that systemic administration of OV remains challenging [45]. Several factors may explain the limited systemic efficacy of the PD strains. First, our biodistribution data indicate that only minimal amounts of virus reached the tumor tissue after i.v. injection. This may reflect rapid inactivation of viruses when the virus is injected into the bloodstream, a process that occurs via binding to virus-neutralizing antibodies or complement factors and can be mediated by nonspecific antiviral mechanisms [46]. In addition, the liver, as the central filtering organ in the body, further contributes to pronounced viral clearance through Kupffer cells [47]. In our investigations, pre-existing CVB3 antibodies could largely be excluded as a cause of the limited efficacy of PD-H and PD-622TS, since the mice had no prior exposure to PD-H or PD-622TS. Instead, sequestration of the virus by immune cells appears to be of particular relevance. This is supported by our findings showing that substantial amounts of CVB3 VP-1 and replicating PD-H and PD-622TS could be found in immune cells of the spleen. The importance of the latter is supported by previous investigations demonstrating that there is direct interaction of CVB3 with immune cells in the splenic compartment [48]. It is also supported by our own investigation demonstrating that PD-H is able to infect immune cells [12]. Furthermore, a high percentage of the virus might be sequestered to normal organs, such as the pancreas, heart, liver, and lungs, in which we found virus at low to moderate titers. In addition, the heparan-sulphate-dependent entry mechanism of PD-H and PD-622TS, although advantageous for certain tumor types, may limit viral dissemination in vivo due to interactions with extracellular matrix components or serum factors [49]. Endothelial cells of the vessels, for example, express 6-O-heparansulfates [50] and are able to be infected by PD-H in vivo [12]. Therefore, sequestration of the viruses to endothelial cells may also contribute to the failing infective ability of the tumor after i.v. administration of PD-H and PD-622TS.

Second, the limited oncolytic activity of PD-H and PD-622TS could also be directly related to the specific architecture of solid tumors, particularly the presence of anatomical barriers. The often tight extracellular matrix, low vascularization, and high interstitial pressure counteract penetration of the virus within the tumor tissue [17,51,52]. In contrast, direct i.t. injection leads to local destruction of the tumor microenvironment, thereby improving infection of tumor cells and the spread of the virus within the tumor tissue. Consistent with this, we observed that i.t. PD-H injection in subcutaneous Colon-26 tumors, as we carried out previously, resulted in virus detection and moderate virus titers in four of five animals 3 days after virus administration [16]. In contrast, PD-H was only detected in one of six animals after i.v. injection, and, moreover, the virus titer was approximately 1000-fold lower than it was after i.t. injection. As a consequence, PD-H needs additional engineering to enhance systemic tumor homing and reduce viral clearance, thus making it suitable for systemic application. For example, a recently published study demonstrated that oCVB3 packaged in exosomes and coupled to a tumor-targeting aptamer was able to successfully treat breast tumors in vivo following systemic administration [53]. The exosome carrier might also be suitable for protecting i.v.-administered PD-H or PD-622TS and increasing the infection of colorectal tumors with the virus. However, this still needs to be confirmed in future studies.

Systemic delivery of PD-H resulted in detectable viral loads in multiple organs and was accompanied by pathological alterations in the pancreas and heart in the mice. This observation contrasts with our recent data obtained after i.t. administration of PD-H into subcutaneous Colon-26 tumors, wherein organ infection and tissue damage were not detected [16]. This demonstrates that the route of virus administration has a substantial impact on the safety profile of PD-H. Intravenous delivery appears to be associated with a greater risk than i.t. application, probably because viral dissemination via the bloodstream allows PD-H to reach CVB3-susceptible organs such as the heart and pancreas rapidly and directly. Nonetheless, compared with infections caused by i.v.-injected wild-type CVB3 [19], the organ damage induced by i.v.-injected PD-H is distinctly reduced. In fact, here, we found that the viral titers in the organs were low, pathological alterations were mild, and only a minority of the animals were affected. In addition, long-term analyses showed that organ damage found early after infection healed completely. The reasons for PD-H attenuation in normal tissues have not been explored. However, they may also be related to the unique tropism [12,49,54]. Notably, the occurrence of organ pathology in only a subset of the PD-H-treated animals highlights interindividual differences in host susceptibility to the virus. This variability observed in mice may have implications for the use of PD-H in humans, as certain patients could likewise be more susceptible to PD-H than others.

To further enhance the overall safety of PD-H, we developed the new microRNA-regulated PD-H derivatives PD-622TS and PD-145TS. Both viruses exhibited markedly improved safety profiles after i.v. administration compared to PD-H, with PD-622TS performing slightly better in the direct comparison. In contrast to our previously generated PD-H variant PD-375TS, which expresses miR-TS of the pancreas-specific expressed miR-375 [16], both new microRNA-regulated viruses contain miR-TS that enables inhibition of viral replication not only in the pancreas but also in several other organs that are susceptible to CVB3 infection. In this context, it is important to note that both viruses maintained robust replication kinetics and cytotoxic activity in the murine colorectal cancer cell lines Colon-26 and CT-26Luc. PD-145TS, however, displayed slightly reduced cytotoxicity compared to PD-622TS, most likely attributable to the relatively high endogenous levels of miR-145 in both cell lines. In contrast to the murine models, all human colorectal cancer cell lines analyzed exhibited low miR-145 expression, suggesting that PD-145TS may also be appropriate for therapeutic application in human patients with colorectal cancer. Nevertheless, based on the findings presented here, an evaluation of miR-145 levels in patient tumor samples prior to treatment should be conducted. This assessment could help avoid the potentially ineffective administration of PD-145TS to patients with elevated miR-145 levels.

Beyond the improved acute safety profile, further design considerations are essential for the continued development of microRNA-regulated oncolytic RNA viruses. The inherent genetic instability of RNA virus replication may lead to the mutation or loss of inserted miR-TS, which can compromise virus attenuation in normal tissue. To address this risk, multiple copies of a given miR-TS can be incorporated into the viral genome, as demonstrated previously [20,21,23] and in the present study. This approach increases the probability that at least one miR-TS copy remains functionally intact even in the presence of partial sequence alterations [20]. Although no evidence of the loss of microRNA-mediated regulation was observed within the experimental timeframe of this study, long-term genetic stability remains an important consideration for the future development of oCVB3.

In summary, this study demonstrates that systemic in vivo administration of PD-H results in negligible antitumor activity against colorectal carcinomas and is associated with off-target tissue infection and adverse effects. The newly engineered PD-H derivatives PD-145TS and PD-622TS substantially reduce these side effects and thereby improve the systemic safety profile of the virus. Although therapeutic efficacy after i.v. administration remains constrained, these microRNA-regulated PD-H variants provide a safer platform for further virus engineering. Additional strategies of enhancing tumor delivery and bioavailability—such as carrier-based delivery, exosome-mediated transport, or capsid engineering—will likely be required to allow the viruses to have meaningful systemic antitumor effects.

## Data Availability

Data will be supplied following reasonable requests.
